# The Effects of Pitch Shifts on Delay-Induced Changes in Vocal Sequencing in a Songbird

**DOI:** 10.1523/ENEURO.0254-16.2017

**Published:** 2017-01-20

**Authors:** MacKenzie Wyatt, Emily A. Berthiaume, Conor W. Kelly, Samuel J. Sober

**Affiliations:** Department of Biology, Emory University, Atlanta, GA 30322

**Keywords:** Bengalese finch, birdsong, sensorimotor learning, stuttering

## Abstract

Like human speech, vocal behavior in songbirds depends critically on auditory feedback. In both humans and songbirds, vocal skills are acquired by a process of imitation whereby current vocal production is compared to an acoustic target. Similarly, performance in adulthood relies strongly on auditory feedback, and online manipulations of auditory signals can dramatically alter acoustic production even after vocalizations have been well learned. Artificially delaying auditory feedback can disrupt both speech and birdsong, and internal delays in auditory feedback have been hypothesized as a cause of vocal dysfluency in persons who stutter. Furthermore, in both song and speech, online shifts of the pitch (fundamental frequency) of auditory feedback lead to compensatory changes in vocal pitch for small perturbations, but larger pitch shifts produce smaller changes in vocal output. Intriguingly, large pitch shifts can partially restore normal speech in some dysfluent speakers, suggesting that the effects of auditory feedback delays might be ameliorated by online pitch manipulations. Although birdsong provides a promising model system for understanding speech production, the interactions between sensory feedback delays and pitch shifts have not yet been assessed in songbirds. To investigate this, we asked whether the addition of a pitch shift modulates delay-induced changes in Bengalese finch song, hypothesizing that pitch shifts would reduce the effects of feedback delays. Compared with the effects of delays alone, combined delays and pitch shifts resulted in a significant reduction in behavioral changes in one type of sequencing (branch points) but not another (distribution of repeated syllables).

## Significance Statement

Vocal behavior depends critically on an organism’s ability to monitor the sound of its own voice (“auditory feedback”). Studies of both humans and songbirds have demonstrated that successful vocal performance depends critically on the quality and timing of such feedback; however, the interaction between vocal acoustics and the timing of auditory feedback is unclear. Here we used songbirds to examine this interaction by measuring vocal performance during delays and distortions (pitch shifts) of auditory feedback.

## Introduction

**Figure 1.**
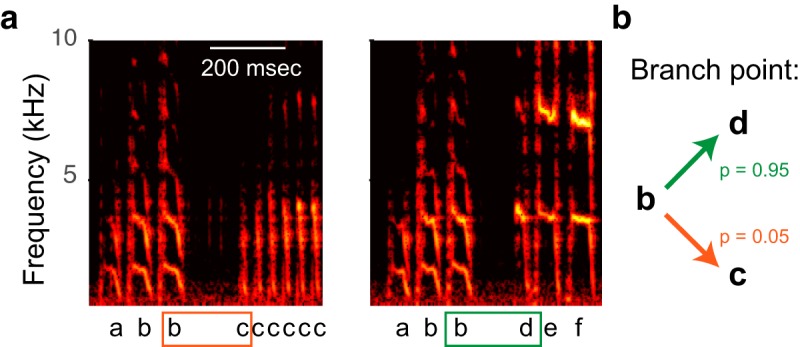
Sequence variation in birdsong. ***A***, Spectrographic representations show the power (heat map) at each acoustic frequency (vertical axis) as a function of time (horizontal axis). The two spectrograms show excerpts from different times during the same bout of song from a single bird. Labels below the spectrogram indicate different syllables. Orange and green boxes highlight a branch point in which syllable b can be followed by either syllable d or syllable c. ***B***, Schematic quantifies transition probabilities for this branch point.

Learned vocal behaviors depend strongly on auditory feedback. In both birdsong and human speech, adults rely on auditory feedback to detect and correct errors in vocal production. This reliance on auditory information can be demonstrated by manipulating auditory feedback and measuring the effects on vocal output. Complete elimination of auditory feedback by deafening in adulthood leads to dramatic vocal performance deficits (McGarr, 1983; Okanoya and Yamaguchi, 1997; Woolley and Rubel, 1997; Lombardino and Nottebohm, 2000). More subtle manipulations of auditory signals reveal the complex influence of sensory feedback on motor programming. Artificially delaying auditory feedback in human speakers can cause vocal sequencing errors, including unwanted repetitions of consonants and words, in normally fluent speakers (Fairbanks, 1955; Chase, 1958; Yates, 1963). Such results suggest that the sequencing errors observed in persons who stutter might result from disorders of auditory feedback processing (Büchel and Sommer, 2004; Hampton and Weber-Fox, 2008). Intriguingly, artificially delaying auditory feedback is sometimes effective as a treatment for stuttering (Ryan and Van Kirk, 1974; Kalinowski and Stuart, 1996), further linking the dependence of vocal sequencing on the timing of auditory feedback and emphasizing the complex relationship between sensory feedback and speech production. Analogously, studies of birdsong have shown that perturbations of auditory feedback timing can degrade vocal production. Delayed playbacks of a bird’s own syllable during singing leads to song degradation after chronic exposure in zebra finches (Leonardo and Konishi, 1999; Cynx and von Rad, 2001). In Bengalese finches, a species whose song contains “branch points” where vocal sequencing is probabilistic rather than fixed (Fig. 1), acute changes in vocal sequencing can result from delayed playbacks of a bird’s own song syllable while singing (Sakata and Brainard, 2006). The similarities of these results across species suggest songbirds as a promising animal model for disorders of human speech production.

Other studies in songbirds and humans have explored how the brain uses the acoustic structure of auditory feedback (as distinct from the timing of feedback) to calibrate vocal performance. In both songbirds and humans, manipulations of the fundamental frequency (which we refer to here as pitch) of auditory feedback evoke compensatory responses, for example by increasing the pitch of vocal output in response to a decrease in the pitch of online auditory feedback (Jones and Munhall, 2000; Sober and Brainard, 2012; Hoffmann and Sober, 2014). Notably, vocal pitch changes in birdsong and formant changes in human speech are most robust for smaller shifts in auditory feedback, with larger shifts evoking little or no change in vocal output (Burnett et al., 1998; Liu and Larson, 2007; MacDonald et al., 2010; Katseff et al., 2012; Sober and Brainard, 2012), suggesting that during large pitch shifts, the brain relies less on auditory feedback to influence ongoing vocal behavior. Intriguingly, large (half an octave) pitch shifts cause an increase in speech fluency in some persons who stutter (Kalinowski et al. 1993; Natke et al., 2001), suggesting an interplay between the acoustic structure of auditory feedback and the sequencing of vocal motor commands. However, the relationship between the acoustics and timing of vocal feedback, and their influence on vocal output, remain poorly understood. By examining the interactions between the acoustic structure and timing of auditory feedback in songbirds, we hope to better understand the interaction between sensory and vocal motor signals. Such an understanding will position the songbird as an animal model in which neural activity during normal and disordered vocal production might be examined, and the neural mechanisms by which auditory feedback delays and pitch shifts can improve vocal performance in persons who stutter might be understood.

We used Bengalese finches to investigate whether delay-induced changes in vocal production were influenced by alterations in the pitch of auditory feedback. Although the effects of auditory feedback delays on song have been tested previously using song-triggered playbacks of previously recorded songs, technical challenges have prevented the use of continuous delayed feedback. We overcame this obstacle using miniaturized headphones (see Methods), which provided continuous, delayed feedback in real time (Hoffmann et al., 2012). Experimental conditions included a null condition (no delay or pitch manipulation), delayed auditory feedback (DAF) without any pitch shift, and a condition in which auditory feedback was both delayed and pitch shifted (DAF+PS). We predicted that, in agreement with prior findings using playbacks of short segments of song (Sakata and Brainard, 2006), delayed feedback would induce changes in the syllable transition probabilities. In particular, we hypothesized that, as reported by Sakata and Brainard (2006), delayed feedback would result in the most common transition (the “primary” transition) becoming less prevalent and the nonprimary transition becoming more common. We further hypothesized that the large pitch shift in the DAF+PS condition would reduce the magnitude of the changes observed in the DAF condition.

## Materials and Methods

Four adult (>100 d old) male Bengalese finches (*Lonchura striata* var. *domestica*) obtained from an outside vendor were used as the experimental subjects. During experiments, birds were housed individually in an isolated sound-attenuating chamber, and all song was undirected (i.e., produced in the absence of female birds). The light/dark cycle was maintained for 14:10 h, with lights on beginning at 7 am and ending at 9 pm. All procedures were approved by the Emory University Institutional Animal Care and Use Committee.

### Experimental procedure

**Figure 2.**
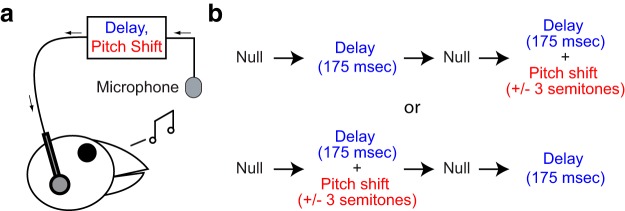
Experimental design. ***A***, Auditory feedback manipulated using miniaturized headphones. A microphone transmits a bird’s vocalizations to online sound processing hardware, which is used to introduce a 175-ms delay (with or without a ±3 semitone pitch shift). ***B***, Schedule of experimental conditions. Each bird was exposed to the delayed feedback alone (DAF) and delayed feedback plus a pitch shift (DAF+PS). The order of these conditions, as well as the direction of the pitch shift, was randomized across subjects. Before each DAF or DAF+PS epoch, birds sang in a null epoch free of delays or pitch shifts. The four experimental epochs lasted 5 d each.

Miniature, lightweight headphones were custom-built out of lightweight carbon fiber and custom-fitted to each bird’s head (Hoffmann et al., 2012). A condenser microphone in the bird’s cage (Fig. 2*A*) captured the bird’s acoustic output, which was routed to online sound-processing hardware (Eventide H7600), which provided perturbations of the pitch and/or timing of auditory feedback in real time. This manipulated feedback was then relayed to miniaturized speakers (EH-7157-000, Knowles) inside the headphones. In addition to the speakers, the headphones apparatus included a miniaturized microphone (EM-3046, Knowles) placed between the speaker and the opening of one of the ear canals. This microphone allowed us to monitor the performance of the headphones apparatus. The miniaturized microphone was used to calibrate the system such that the acoustic signal played through the headphones speakers was ∼2 log units greater than auditory feedback leaking through the carbon fiber frame. The headphones therefore shielded the birds’ airborne vocalizations, allowing the altered feedback to replace the natural version. As described in detail below, auditory feedback conditions included a null condition in which no manipulation was introduced, a DAF condition in which auditory feedback was delayed by 175 ms, or a DAF+PS condition in which both a 175-ms delay and an upward or downward pitch shift were applied simultaneously. As detailed previously, the sound-processing hardware relayed the online acoustic signal to the headphones with a minimal delay (i.e., when auditory feedback was not being intentionally delayed) of ∼10 ms, a delay which does not in itself evoke any measurable changes in vocal behavior (Sober and Brainard, 2009; Hoffmann et al., 2012; Kelly and Sober, 2014). Note that subjects might receive some unmanipulated auditory feedback via bone conduction, although such a scenario is very unlikely to account for our results (see Discussion).

The experimental design is outlined in Fig. 2*B*. Once birds habituated to the headphones, all birds sang during a period with zero pitch shift or delay for 5 d. After this null period, in the example shown at top in Fig. 2*B*, the birds’ auditory output was altered with delayed auditory feedback (DAF block) for 5 d. The birds heard their natural vocalizations at 175-ms delay relative to output. We selected this delay magnitude after preliminary studies in two birds (not used in the present study) suggested that this delay consistently evoked changes in vocal sequence; however, different delay values were not tested systematically. After the altered auditory feedback block, birds were subjected to a second 5-d null period of singing with zero pitch shift and no introduced delay. The birds were then subjected to a delayed auditory feedback and pitch shift block (DAF+PS) lasting 5 d. During the DAF+PS block, delayed feedback at 175 ms was concurrently pitch-shifted up or down by three semitones. Both the sign of the pitch shift (i.e., upward or downward) in the DAF+PS condition and the order of the DAF and DAF+PS blocks were varied across birds to counterbalance any learning order effects.

The magnitude of the pitch shift (± 3 semitones) was selected based on prior research on the effects of auditory manipulations on both songbirds and humans. As described in the Introduction, smaller shifts in the pitch or formant structure of auditory feedback evoke robust changes in vocal output. Larger shifts, on the other hand, evoke less robust changes in the pitch of human speech (Burnett et al., 1998; Liu and Larson, 2007). Importantly, large pitch shifts have been shown to reduce stuttering in human speech (Kalinowski et al., 1993; Natke et al., 2001). Because one motivation for our songbird studies was to develop an animal model of how the acoustic and temporal features of auditory feedback interact during human speech, we chose a pitch shift magnitude (± 3 semitones) that has been shown previously to be too large to evoke changes in vocal pitch in Bengalese finches, although smaller shifts do evoke robust pitch changes in song (Sober and Brainard, 2012). Our selection of this pitch shift magnitude therefore represents an attempt to maximize the correspondence between our work in songbirds and prior studies of speech.

### Measuring song syntax features

As in previous uses of the headphones paradigm (Sober and Brainard, 2009), we analyzed songs produced during a fixed time window (here, 8 am to noon). In cases in which birds produced more than 60 bouts of song during this interval, we used only 60 bouts (spaced evenly across the interval) in the analysis. Syllable onsets and offsets were determined using an amplitude threshold, and song syllables were assigned arbitrary labels (e.g., a–f in Fig. 1) by visual inspection. Note that the use of the same letters for labeling syllables across different birds does not indicate acoustic similarities between the birds’ syllables.

We examined syllable sequencing in two contexts: branch points and repeated syllables. At a branch point, a single syllable can be followed by multiple different syllables. Such sequence variability is a hallmark of Bengalese finch song (Okanoya, 2004; Wohlgemuth et al., 2010; Matheson and Sakata, 2015), and branch point probabilities are actively maintained during vocal learning (Warren et al., 2012). At each branch point, we quantified the probability of each transition (e.g., Fig. 1*B*). We used a *z*-test for proportions to compare probabilities from altered auditory feedback conditions to the null condition immediately preceding it. For a group analysis of the effects of DAF and DAF+PS on branch point probabilities across birds, we used a one-sided Wilcoxon signed rank test to evaluate our hypothesis that the effects of delayed auditory feedback would be reduced if the delay were performed in the presence of a pitch shift. We performed this statistical test only on changes in the probability of the primary (most common) transition, for two reasons. First, and most importantly, the probabilities of primary and nonprimary transitions at a single branch point are not independent. For example, if there are only two transitions and one increases by 10%, then the other must decrease by the same amount, so it would be incorrect to consider changes in two transitions at a single branch points as separate measurements. Second, we focused on the primary transition to evaluate our hypothesis that delayed auditory feedback (in both the DAF and DAF+PS condition) would lead to a reduction in the probability of the primary transition, as observed previously in a similar experiment (Sakata and Brainard, 2006). In total, the four birds used in our studies yielded a total of nine branch points (one to four per bird), consisting of four cases in which one syllable could be followed by one of two different syllables, and five cases in which one syllable could be followed by one of three different syllables.

**Figure 3.**
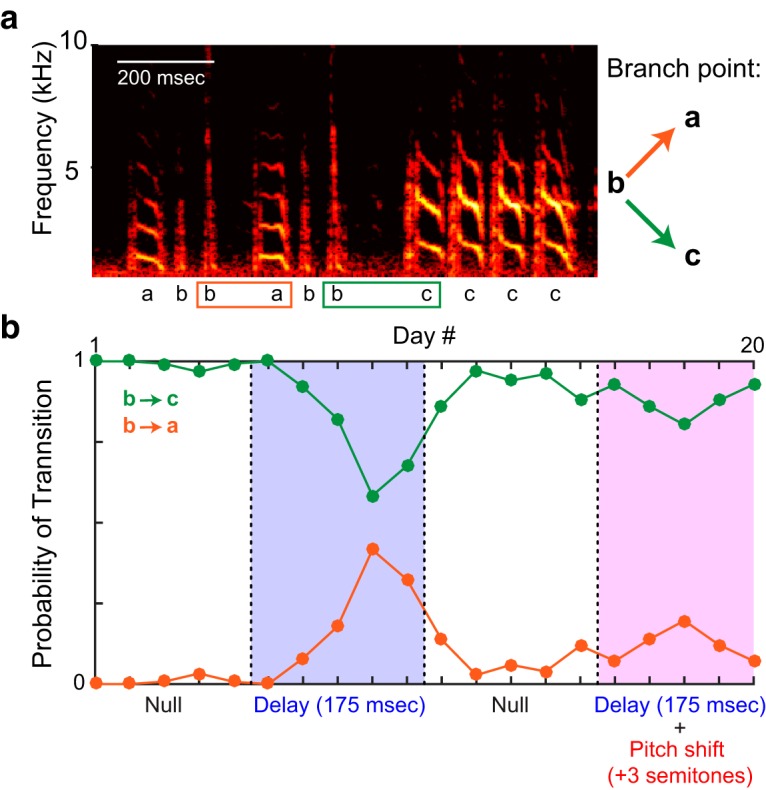
Effects of auditory feedback manipulations on branch point probabilities. ***A***, One branch point (in which syllable b can be followed by either syllable a or c), of nine total branch points in our dataset. Spectrogram plotting conventions as in Fig. 1*A*. ***B***, In this experiment, after a null period of unmanipulated auditory feedback, the bird experienced DAF followed by another period of unmanipulated feedback, followed by DAF+PS. Green and orange traces show the probability of the b-to-c and b-to-a transitions, respectively.

Bengalese finches also commonly produce repeated syllables (e.g., syllable c in Fig. 3*A*), which are produced multiple times in succession. We quantified the distribution of repeat numbers for each repeated syllable in each tested auditory feedback condition (for example, the excerpt of song shown in Fig. 3*A* contains a case in which syllable c is repeated four times). We used a Kolmogorov–Smirnov test to determine whether the repeat distributions of individual syllables differed significantly across feedback conditions, comparing the repeat distribution in the DAF or DAF+PS condition with that of the null period immediately preceding it. As in the analysis of branch point probabilities, we used a Wilcoxon signed-rank test to determine whether the change in mean repeat number from null to DAF was significantly larger than the changes induced by the DAF+PS. In all statistical tests of both branch points and repeated syllables, we used data from only the last 3 d of each auditory feedback condition. The four birds examined yielded a total of 20 repeated syllables (three to nine per bird). In all analyses described above, for each branch point or repeated syllable, we combined data across the last 3 d of the 5-d feedback epoch (null, DAF, or DAF+PS) when computing the effects of each feedback condition.

## Results

**Figure 4.**
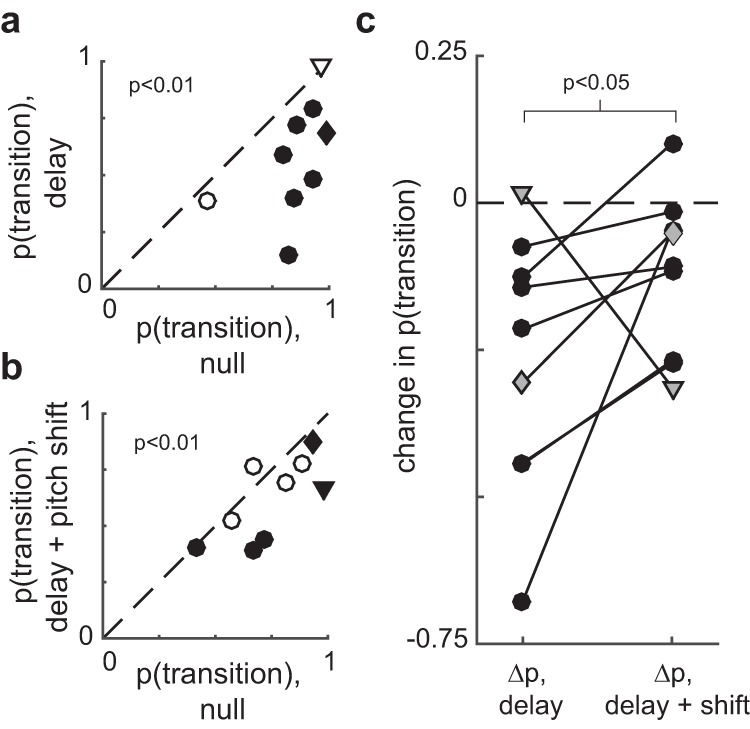
Effects of auditory feedback manipulations on branch points (group data). ***A***, Probability of the primary (i.e., most common; see Methods) transition in the null versus DAF conditions. Filled circles indicate that the difference between each probability in the null and DAF condition was statistically significant (*p* < 0.05, *z*-test for proportions). Across all cases, transition probabilities significantly decreased as a result of DAF (*p* < 0.01, one-sided Wilcoxon signed rank test). ***B***, Transition probabilities in the null versus DAF+PS condition. Other plotting conventions as in ***A***. ***A***, Comparison of the change in transition probability induced by the DAF and DAF+PS conditions. As hypothesized, the change in probability was significantly smaller in the DAF+PS condition (*p* < 0.05, one-sided Wilcoxon signed rank test). In all plots, diamond symbols indicate data from the example shown in Fig. 3, and triangle symbols indicate data from the example shown in Fig. 5.

As hypothesized, delaying auditory feedback often induced changes in syllable sequencing. Fig. 3 shows data from one branch point. In the first null period, syllable b was followed by syllable c >95% of the time (green trace, Fig. 3*B*), and was therefore the primary transition at that branch point (see Methods). Syllable b was followed by syllable a <5% of the time (orange trace, Fig. 3*B*). During the DAF condition (blue shaded region, Fig. 3*B*), transition probabilities gradually shifted, with the b-to-c transition becoming less common and the b-to-a transition becoming more common. Fig. 4*A* summarizes the effects of DAF on transition probabilities across all branch points examined. Our dataset contained nine branch points with a mean of 314 iterations (range 71–739) of each branching sequence. In seven of nine cases, delayed feedback led to a significant reduction in the probability of the primary transition (Fig. 4*A*, filled symbols, *p* < 0.05, *z*-test for proportions). When considered as a group, transition probabilities decreased significantly as a result of DAF being applied (*p* < 0.01, one-sided Wilcoxon signed rank test).

In the example shown in Fig. 3, the DAF+PS condition (pink shaded region) had a similar, but smaller, effect on syllable sequencing than DAF, with the b-to-c transition becoming slightly less prevalent in the DAF+PS epoch compared to the preceding null period. Fig. 4*B* shows the effects of DAF+PS across transition points. Similar to the DAF condition (Fig. 4*A*), DAF+PS induced significant changes in most cases (Fig 4*B*, filled symbols, *p* < 0.05, *z*-test for proportions) and as a group exhibited a significant reduction in the probability of the primary transition (*p* < 0.01, one-sided Wilcoxon signed rank test).

We then asked whether, consistent with our hypothesis, the changes induced by DAF+PS were smaller than those induced by DAF alone. Fig. 4*C* compares the change in transition probability induced by DAF (Δ*p*, delay) with that induced by DAF+PS (Δ*p*, delay + shift). As hypothesized, the effects of DAF+PS were significantly smaller (*p* < 0.05, one-sided Wilcoxon signed rank test).

**Figure 5.**
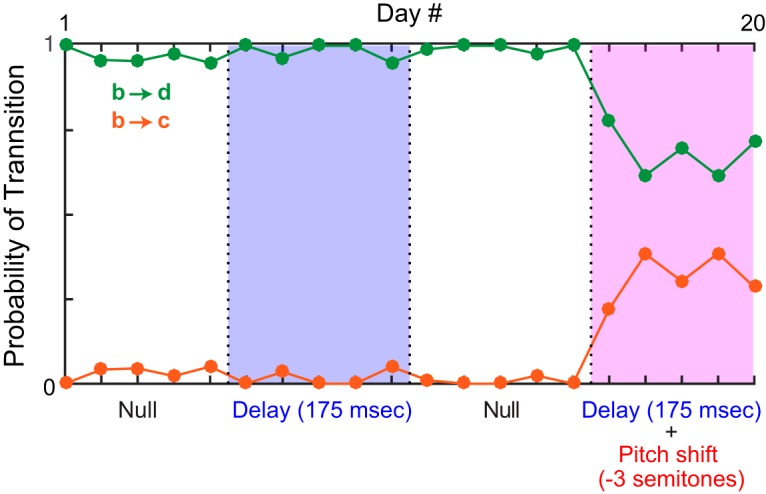
Additional example of effects of feedback manipulations on branch point probabilities. Shown is data from one branch point (of nine total branch points in our dataset). Plotting conventions as in Fig. 3. Data are from the branch point also shown in Fig. 1.

Notably, although overall DAF+PS produced significantly smaller changes in transition probability than DAF, in one case (triangle symbols in Fig. 4), much larger changes were observed in the DAF+PS condition. Data from this branch point are shown in Fig. 5. Therefore, it is important to emphasize that although the group analysis demonstrated smaller changes once pitch shifts were added to delays, the opposite was seen in one individual case.

**Figure 6.**
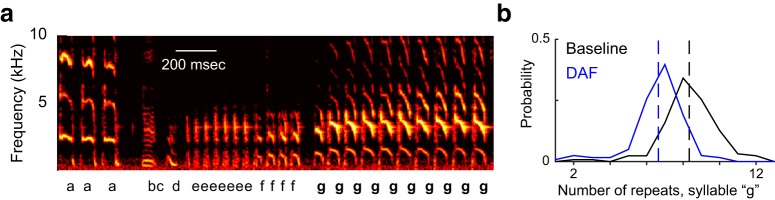
Effects of auditory feedback manipulations on repeat lengths. ***A***, Spectrogram shows excerpt of a song that contains three repeated syllables (e, f, and g). In this excerpt, syllable g is repeated 10 times. Spectrogram plotting conventions as in Fig. 1*A*. ***B***, Distribution of repeat lengths of syllable g in the null condition (black solid line) and DAF condition (blue solid line). Dashed lines show the mean of each distribution.

**Figure 7.**
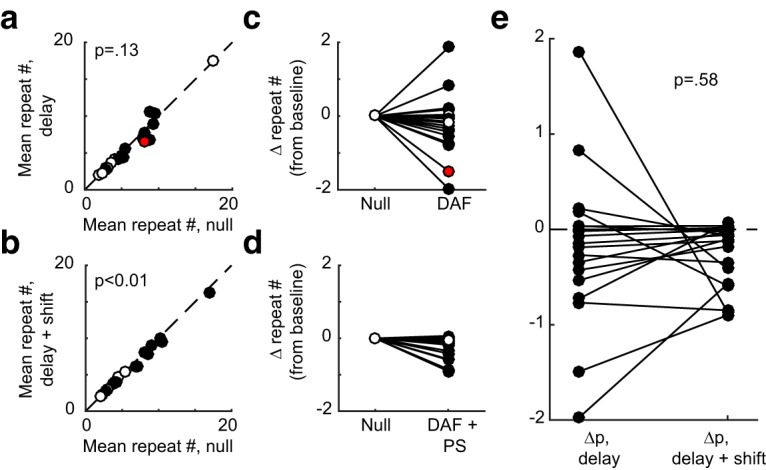
Effects of auditory feedback manipulations on repeat lengths (group data). ***A***, Mean repeat length in the null versus DAF conditions. Filled circles indicate that the difference between the repeat distribution in the null and DAF condition was statistically significant (*p* < 0.05, two-sample Kolmogorov–Smirnov test). Across all cases, mean repeat numbers did not differ significantly as a result of DAF (*p* = 0.13, one-sided Wilcoxon signed rank test). ***B***, Mean repeat lengths in the null versus DAF+PS condition. Other plotting conventions as in ***A***. ***C***, ***D***, The same data as panels ***A*** and ***B***, respectively, displayed at the absolute difference in mean repeat number. Red dot in ***A*** and ***C*** corresponds to the data shown in Fig. 6. ***E***, Comparison of the change in mean repeat number induced by the DAF and DAF+PS conditions. No significant difference was detected between the changes in repeat number in the DAF and DAF+PS conditions (*p* = 0.65, one-sided Wilcoxon signed rank test).

We also examined the effect of auditory feedback manipulations on the distribution of repeated syllables (Fig. 6*A* shows an example containing four different repeated syllables). Fig. 6*B* shows an example from our dataset in which DAF induces a significant change in the distribution of repeats of syllable g (*p* < 0.05, Kolmogorov–Smirnov test). As shown in Fig. 7*A*, DAF frequently led to significant changes in repeat distribution (filled symbols), although there was no significant bias toward increases or decreases in mean repeat number (*p* = 0.13, two-sided Wilcoxon signed rank test). The DAF+PS condition (Fig. 7*B*) also induced significant changes in the repeat distribution in many cases. Interestingly, in the majority of these cases (16/20), DAF+PS reduced the mean number of repeats (*p* < 0.01, two-sided Wilcoxon signed rank test). Fig. 7*C*, *D* shows the same data as Fig. 7*A*, *B*, respectively, but represented as a change in repeat number between the null and DAF/DAF+PS conditions.

We next evaluated our hypothesis that the DAF+PS condition would evoke smaller changes in repeat number than the DAF condition. Comparing these changes (Fig. 7*E*) did not reveal any significant difference between the two conditions (*p* = 0.65, two-sided Wilcoxon signed rank test). We further asked whether any differences existed between the data from the two conditions shown in Fig. 7*E* by performing a two-sample Kolmogorov–Smirnov test, which similarly failed to detect any significant difference (*p* = 0.77). Therefore, although in individual cases both branch point probabilities (Fig. 4*A*, *B*) and repeat number (Fig. 7*A*, *B*) were often significantly modulated by DAF or DAF+PS, the effects of these two alterations of auditory feedback differed significantly only for branch point probabilities (Fig. 4*C*) and not for the distribution of repeated syllables (Fig. 7*E*).

## Discussion

Manipulation of auditory feedback induced robust sequence changes in the song of adult Bengalese finches. As hypothesized, both DAF and DAF+PS induced changes in transition probabilities and repeat length distributions in a substantial number of individual cases (filled symbols, Figs. 4*A*, *B* and 7*A*, *B*). At branch points, both feedback manipulations induced a reduction in the probability of the primary transition (Fig. 4*A*, *B*). In contrast, whereas DAF did not significantly bias changes in mean repeat number upward or downward (Fig. 7*A*, *C*), DAF+PS caused a reduction in mean repeat number in a significant fraction of cases (Fig. 7*B*, *D*). Together, these results demonstrate that continuous, real-time manipulation of auditory feedback can strongly modulate vocal performance and that in some contexts, the addition of a pitch shift can significantly reduce the vocal changes induced by auditory feedback delays.

A number of studies have examined the consequences of subjecting birds to song-triggered playbacks of (previously recorded) samples of the bird’s own song, a manipulation that approximates delayed auditory feedback. In contrast, our technique uses miniaturized headphones to continuously stream manipulated auditory feedback. It is possible that this methodological difference accounts for some apparent discrepancies between our results in the DAF condition and prior findings. Notably, Sakata and Brainard (2006) used playbacks of one to three song syllables at specific times during Bengalese finch songs. Similar to our findings, they found that playbacks targeted to branch points reduced the probability of the primary transition (Sakata and Brainard, 2006). In contrast to our findings, however, they noted that the effects of feedback manipulation were observed very soon after the manipulation was introduced and did not increase with continued exposure, whereas in many of our experiments (Figs. 3*B* and 5), the magnitude of DAF effects on sequence grew steadily over the first few days of exposure. Although the variation in behavioral effects might reflect the different methods of altering auditory feedback, additional studies would be required to isolate the effects of continuous, real-time feedback (our study) versus intermittent, prerecorded feedback (Sakata and Brainard, 2006) from other methodological differences between the two studies, including the total time of exposure to altered feedback and the magnitude of the feedback delay.

The headphones apparatus greatly attenuates airborne transmission of a bird’s vocalization, replacing it with the manipulated version played through the headphone speakers. However, as discussed elsewhere, subjects might receive unmanipulated acoustic feedback via bone conduction, in which sound is transmitted via body tissues rather than air (Sober and Brainard, 2009). Although we cannot rule out some influence of bone conduction, we note that this factor presumably applies in both the DAF and DAF+PS conditions, and therefore seems unlikely to account for the differing effects of these results. We further note that potential bone conduction signals are only one of several sensory modalities that can convey unmanipulated feedback, with proprioceptive/somatosensory systems additionally providing information about the birds’ actual motor output.

Our findings highlight the importance of the characteristics of auditory feedback on vocal behavior. As shown in Fig. 4*C*, DAF elicited significantly larger changes in branch point transition probability than did DAF+PS, as hypothesized. This finding is significant for two reasons. First, it parallels similar findings in persons who stutter. The vocal sequencing errors that typify stuttering can be reduced by the application of an online pitch shift (Kalinowski et al., 1993; Natke et al., 2001; Büchel and Sommer, 2004). Our analogous finding in songbirds (i.e., that delay-induced sequencing changes can be partly reversed by pitch shifts) suggests that songbirds might be used as an animal model of how temporal and acoustic properties of auditory feedback might be manipulated to enhance the fluency of human speech. Second, our findings suggest that pitch shifts reduce songbirds’ reliance on auditory feedback when sequencing vocal behavior. A prior study employing pitch shifts, but not delays, found that while smaller (±0.5 or 1.0 semitone) pitch shifts evoke compensatory changes in vocal pitch, ±3.0 semitone pitch shifts did not evoke robust changes in vocal acoustics (Sober and Brainard, 2012). The present findings suggest that pitch-shifted auditory feedback is similarly disregarded when animals program upcoming vocal sequences.

Our analyses did not reveal any significant difference in the effects on repeat number evoked by DAF and DAF+PS. Although further refinements of our technique, such as testing of other delay magnitudes, might reveal such a difference, it is also possible that these two forms of variable sequencing (branch points and repeated syllables) differ in their reliance on the acoustic structure of auditory feedback (Wittenbach et al., 2015). Future studies could examine this possibility by investigating the effects of sensory perturbations on behavior and neural activity during vocal production.

## Synthesis

Reviewing Editor: Tatyana Sharpee, The Salk Institute for Biological Studies Decisions are customarily a result of the Reviewing Editor and the peer reviewers coming together and discussing their recommendations until a consensus is reached. When revisions are invited, a fact-based synthesis statement explaining their decision and outlining what is needed to prepare a revision will be listed below. The following reviewer(s) agreed to reveal their identity: Wan-Chun Liu.

Both Reviewers appreciated the importance of this study and the reported results demonstrating that delayed feedback can be compensated by additional frequency shifts. The main points that would be good to revise are (1) to add an explanation as to why these particular values of frequency shifts/delays were chosen and (2) to strengthen if possible statitistical analyses. For example, one could check if removing some points still produces statistically significant results.

Reviewer 1:

Both birds and humans produce learned vocalizations that rely on real-time auditory feedback. In both, delayed auditory feedback interferes with vocal production, and internal auditory delay has been hypothesized to be the cause of vocal dysfluency.

Interestingly, it has been shown that a large shift in pitch of the auditory feedback restores normal speech in some dysfluent speakers. To explore the roles played by auditory feedback in shaping vocal output, it will be useful to identify an animal model that exhibits a similar dependency on auditory feedback. In this study, the authors discovered that in Bengalese finches, shifting the pitch of the delayed auditory feedback rescues syntactical deterioration that is elicited in response to delayed auditory feedback alone. Although the study is interesting and easy to read, I have several comments.

Introduction

• Although it is interesting to contrast humans and songbirds, it is not immediately clear from reading the introduction why the study was conducted in birds, and not in humans. I assume that the goal of the authors is to carry out a neurophysiological study to understand the function of the auditory feedback on vocal production which cannot be easily carried out in humans. The reason for conducting a study in Bengalese finches should be clarified.

Methods

• The issues of sound conduction via bone should be mentioned in the Methods section.

• For null treatment, 10msec delay is very slow. Did the authors observe any difference between intact song (without any headphones) and null treatment songs.

• For syllable repetition, the parameter used for Wilcoxon signed rank test is not stated. Only in the Results section does the reader find out that the authors used mean syllable repeat number.

• Although the results appear to be consistent with what was predicted from their hypothesis, the sample size is very small. In addition to having only four birds in the study, the songs used for analyses appear very small (20 total syllables from 4 birds, 3-9 per bird, 9 total branch point from 4 birds, 1-4 per bird). Do the birds sing very little with the earphones?

The authors used two layers of statistical tests in this study. First, they tested whether branch point probability and syllable repetition number changed depending on the experimental treatment. Second, they grouped the animals and asked whether these two parameters showed significant change in response to the experimental treatments. Although the second test addresses a question that is of importance (whether Bengalese finches, in general, respond to experimental treatment by changing their vocal behavior), the first test addresses whether a particular branch point or syllable they found in songs of four birds used in the study changed in response to treatment. . In addition, the z-test used for the branch point probability is a parametric test, and appears to be inappropriate in this study with a presumably small sample size (although the maximum number of bouts used was mentioned, it is not clear what the real sample size was for each analysis).

Reviewer 2

This is an interesting study as the authors demonstrate the effect of manipulation of auditory feedback on adult song performance. The authors manipulated both temporal and acoustic structure of auditory feedback and found that the combined effect causes less interference on the song performance. Interestingly, the results seem to be consistently with human studies, as the manipulation of the delayed and fundamental frequency of auditory feedback induce compensatory responses, which may provide important insight about the neurological mechanism of speech disorder.

One of the strength in this work is that the authors use Bengalese finches as an animal model; the species' complex and variable learned vocal sequence offers a good opportunity to test the proposed hypotheses. Additionally, the authors use a miniaturized headphones that provides a better condition for conducting continuous, delayed feedback.

I have a few suggestions that need to be clarified,

1. The introduction provides a general review of delayed auditory feedback and pitch shifts in humans and songbirds, but it does not state why it is important to test the interaction between pitch-shift and delay auditory feedback in this study (the authors only indicate such interaction is unclear).

2. What is the effect of pitch shifts on song in those experimental birds? The two experimental conditions are the delayed feedback alone and delayed feedback/pitch shift. Why not present the pitch shift experiments and see how the song performance is different from the previous studies? This might be important as the methods seem to be different from the previous songbird studies. This experiment can also support the authors' hypothesis regarding the effect of acoustic feedback on humans (as the authors mentioned in the Introduction),

3. The sample size (n=4) is small. And I didn't see the sample size listed in Figure 3 or 5. Is it one individual in each figure? Are these birds genetically closely related (siblings) and shared a similar song repertoire?

4. If there are only 4 individual birds tested (n=4), and one of them showed opposite result (Fig.5), it seems less likely that the non-parametric statistics show significant difference? or perhaps the authors did not explain the statistics well in the methods?

5. It is not clear how the degree of pitch shift (+/- 3 semitones) is determined. Have the authors tested different degree of pitch shift?
